# Population pharmacokinetics of inotuzumab ozogamicin in relapsed/refractory acute lymphoblastic leukemia and non-Hodgkin lymphoma

**DOI:** 10.1007/s10928-018-9614-9

**Published:** 2019-03-11

**Authors:** May Garrett, Ana Ruiz-Garcia, Kourosh Parivar, Brian Hee, Joseph Boni

**Affiliations:** 1 Pfizer Oncology, 10646 Science Center Dr, San Diego, CA 92121 USA; 2 Pfizer Oncology, Collegeville, PA USA

**Keywords:** Inotuzumab ozogamicin, Population pharmacokinetics, Time-dependent clearance, B-cell acute lymphoblastic leukemia, B-cell non-Hodgkin lymphoma

## Abstract

This population pharmacokinetics analysis evaluated the target-mediated drug disposition of inotuzumab ozogamicin (InO) through an empirical time-dependent clearance (CL_t_) term and identified potential covariates that may be important predictors of variability in InO distribution and elimination. This analysis was conducted by pooling data from 2 studies of single-agent InO in patients with relapsed or refractory (R/R) B cell acute lymphoblastic leukemia (ALL), 3 studies of single-agent InO, 5 studies of InO plus rituximab (R-InO), and 1 study of R-InO plus chemotherapy in patients with R/R B-cell non-Hodgkin lymphoma (NHL). Pharmacokinetic data included 8361 InO concentration–time observations that were modeled using nonlinear mixed-effects analysis. Covariate relations were identified using generalized additive modeling on base model parameters and then tested in a stepwise manner via covariate modeling. InO concentration was described with a 2-compartment model with linear and time-dependent clearance components. Based on the final model, baseline body surface area was a covariate of the linear and time-dependent clearance components and volume of distribution in the central compartment; baseline percentage of blasts in the peripheral blood was a covariate of the decay coefficient of the time-dependent clearance term (CL_t_); and concomitant rituximab treatment was a covariate of the linear clearance component (CL_1_). The magnitude of change of each pharmacokinetic parameter due to these covariates was not considered clinically relevant. Therefore, no dose adjustment of InO for the treatment of patients with R/R B-cell ALL or NHL is needed on the basis of selected covariates.

**Electronic supplementary material:**

The online version of this article (10.1007/s10928-018-9614-9) contains supplementary material, which is available to authorized users.

## Introduction

Inotuzumab ozogamicin (InO) is a humanized antibody drug conjugate comprising G544, a IgG4 antibody, with a drug-antibody ratio of 6 [1, 2]. InO targets cluster of differentiation 22 (CD22) and all InO molecules are conjugated to *N*-acetyl-γ-calicheamicin, a potent cytotoxic antibiotic, via an acid-labile 4-(4′-acetylphenoxy) butanoic acid linker [1, 3, 4]. After binding to InO, CD22 is quickly internalized into the lysosomal compartment, where *N*-acetyl-γ-calicheamicin dimethyl hydrazide is released to bind to the minor groove of DNA, leading to double-strand DNA cleavage and apoptosis [1, 2, 5]. A previous study demonstrated the stability of InO in human plasma and serum, with a rate of hydrolysis of 1.5–2% per day [6, 7]. Because CD22 is expressed on the surface of B-cells in > 90% of patients with B-cell malignancies [8, 9], it was assessed as a promising therapeutic target for patients with B-cell cancers [10].

The clinical activity and safety of single-agent InO compared with standard intensive chemotherapy in adults with relapsed or refractory (R/R) CD22+ B-cell acute lymphoblastic leukemia (ALL) was assessed in the phase 3 INO-VATE trial [11]. Results from the study showed that InO was associated with a significantly higher complete remission or complete remission with incomplete hematologic recovery rate (95% confidence interval [CI]) than standard of care (81% [72–88%] vs 29% [21–39%]; *P *< 0.001) and with a significantly higher minimal residual disease negativity rate (95% CI) among responders (78% [68–87%] vs 28% [14–47%]; *P *< 0.001). Moreover, significantly more patients taking InO proceeded to stem cell transplant than those who received standard of care (41% vs 11%; *P *< 0.001). Toxicities were manageable in patients taking InO; hepatic toxicities were more frequent with InO versus standard of care. Based on a study of 234 patients with R/R ALL, the clearance at steady state of InO was 0.0333 L/h and the terminal half-life after 4 cycles was 12.3 days [12]. InO was also assessed previously as either a single agent or in combination with chemotherapy for the treatment of CD22+ B-cell non-Hodgkin lymphoma (NHL). InO is currently approved in the European Union and by the US Food and Drug Administration for adults with R/R B-cell ALL.

Population pharmacokinetic (PK) analysis [13] evaluates the sources of variability of InO disposition, which ultimately determines InO exposure and, thus, is an important component for assessing the efficacy and safety of a drug [13, 14]. Similar to the PK of other monoclonal antibodies [15, 16], InO disposition has been described using an empirical time-dependent clearance (CL_t_), as a reflection of target-mediated disposition. This study aims to investigate the CL_t_ of InO and to identify potential covariates that may be important predictors of variability in InO distribution and elimination, to determine whether InO dose adjustments are needed for certain patients.

## Methods

### Study design and patients

The population PK model was developed using clinical data from 2 studies of single-agent InO in patients with R/R B-cell ALL and 3 studies of single-agent InO, 5 studies of InO plus rituximab, and 1 study of InO plus rituximab and chemotherapy, all in patients with R/R B-cell NHL. In these studies, InO was given by intravenous infusion at dosing regimens of 1.2 to 1.8 mg/m^2^ given over 2 or 3 doses weekly in R/R B-cell ALL or as a single dose (0.4–2.4 mg/m^2^) given on day 1 or 2 of each cycle to patients with R/R B-cell NHL (Online Resource 1).

All studies were conducted in accordance with the principles of the Declaration of Helsinki and the International Conference on Harmonisation Guidelines for Good Clinical Practice. Each study protocol was approved by the ethics committee at participating study centers.

### Pharmacokinetic sampling and bioanalytical methods

Pharmacokinetic samples were collected and analyzed for InO, total calicheamicin (NHL studies only), and unconjugated calicheamicin (*N*-acetyl-γ-calicheamicin dimethyl hydrazide). The population PK analysis presented in this manuscript refers to InO concentrations only because total calicheamicin was only available in the NHL studies, and in most samples (91% for NHL and 98% for ALL) unconjugated calicheamicin concentrations were below the lower limit of quantitation (LLOQ). Bioanalytical methods for PK samples of patients with B-cell ALL were developed and validated by PDD (Richmond, VA, USA) or developed and validated by Pfizer Inc (Groton, CT, USA) and transferred to PPD for revalidation. The serum concentrations of InO were measured using validated high-performance liquid chromatography with tandem mass spectrometry (HPLC/MS/MS) with a LLOQ of 1.0 ng/mL designed to indirectly measure *N*-acetyl-γ-calicheamicin DMH molecules conjugated to the InO antibody. Bioanalytical methods for PK samples of patients with NHL were originally developed and validated by Wyeth Research (Pearl River, NY, USA) and subsequently transferred to PPD and revalidated or were originally developed and validated by PPD. The serum concentrations of InO were measured using a validated enzyme-linked immunosorbent assay (ELISA) method designed to directly measure binding of *N*-acetyl-γ-calicheamicin DHM molecules conjugated to the InO antibody. The LLOQs ranged from 50 to 667 ng/mL depending on the study. Linear range (ranging from 1.00 to 11,200 ng/mL), precision (ranging from ≤ 7.53 to  ≤ 22.6%), and bias of the assays (ranging from − 15.3 to 14.5%) are listed in Online Resource 2.

### Base pharmacokinetic model development

**Fig. 1:**
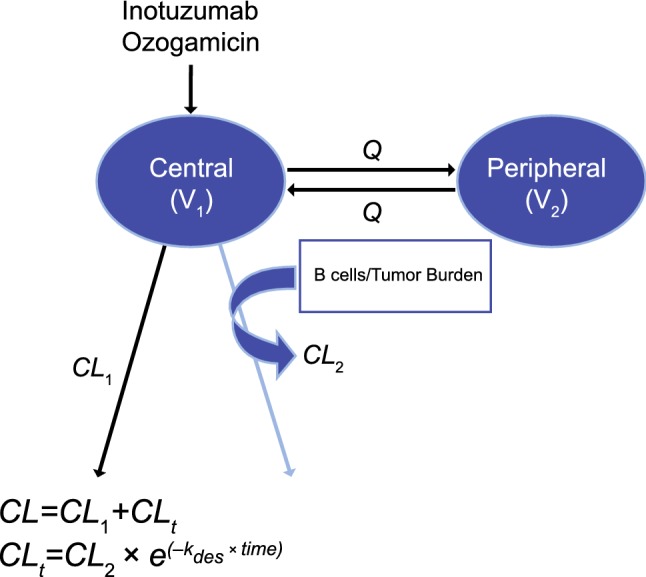
2-Compartment time-dependent clearance model. Refer to Online Resource 3 for further model equations used in NONMEM and Table 2 for definitions of PK parameters

Analysis of the population PK data was conducted using nonlinear mixed-effects modeling (NONMEM 7, level 2.0 and 3.0; ICON Development Solutions, Ellicott City, MD, USA). The PK of monoclonal antibodies are usually described by a 2-compartment model, either linear or with target-mediated disposition. B-cell-targeting monoclonal antibodies have also been shown to exhibit time-dependent clearance, a possible reflection of treatment-related decreases in target B-cell counts over time. Therefore, the initial 2-compartment model was tested using a linear elimination, Michaelis–Menten elimination, and a time-dependent clearance was explored. Both the linear and Michaelis–Menten 2-compartment models were not able to describe the InO concentration–time profile in cancer patients with NHL or ALL. The best model describing the InO concentration–time profile was a 2-compartment model with a linear and time-dependent clearance components (Fig. 1).

In the time-dependent clearance model, clearance (CL) was the sum of the linear clearance CL_1_ and time-dependent clearance (CL_2_) components (CL = CL_1_ + CL_2_). Time-dependent clearance decreased with time as CL_t_ = CL_2 ·_ e^(−kdes · Time)^, where CL_2_ was the initial value of time-dependent clearance and k_des_ was the decay coefficient of time-dependent clearance. The differential equations used in fitting the data were:

$${\text{K}}_{ 10} = {\text{CL}}/{\text{V}}_{ 1}$$

$${\text{K}}_{ 1 2} = {\text{Q}}/{\text{V}}_{ 1}$$

$${\text{K}}_{ 2 1} = {\text{Q}}/{\text{V}}_{ 2}$$

$${\text{C1}} = {\text{A}}\left( 1\right)/{\text{V}}_{ 1}$$

$${\text{CL}}_{\text{t}} = {\text{CL}}_{ 2} \cdot {\text{e}}^{{( - {\text{kdes}}*{\text{Time}})}}$$

$${\text{CL}} = {\text{CL}}_{ 1} + {\text{CL}}_{\text{t}}$$

$${\text{DADT}}\left( 1\right) = - {\text{K}}_{ 1 2} *{\text{A}}\left( 1\right) + {\text{K}}_{ 2 1} *{\text{A}}\left( 2\right) - {\text{K}}_{ 10} *{\text{A}}\left( 1\right)$$

$${\text{DADT}}\left( 2\right) = - {\text{K}}_{ 2 1} *{\text{A}}\left( 2\right) + {\text{K}}_{ 1 2} *{\text{A}}\left( 1\right)$$

The time-dependent clearance component corresponds to the decrease in capacity of the target-mediated clearance pathway, which may be related to CD22 and tumor burden. The linear clearance component is thought to reflect the endogenous catabolic processes of IgG degradation, hence its linearity. In patients with NHL, peripheral B-cells were rapidly depleted after the first dose of InO; however, in patients with ALL, tumor cells persisted for a longer period after treatment, and the capacity of the target is not expected to be saturated right away. Therefore, this model reflected the distinction between these 2 disease populations.

Interindividual random effects were introduced for all structural PK parameters using multiplicative exponential random effects, and the residual error (intraindividual variability) for observations was modeled additively based on log-transformed data.

Prediction-, residual-, and empirical Bayes estimate (EBE)-based diagnostics were performed after each model iteration to ensure the adequacy of the fit. EBE-based diagnostics assessed potential differences in parameters CL_1_ and k_des_ due to the effects of disease, bioanalytical assay method (i.e., ELISA method for NHL and HPLC/MS/MS for ALL), or both. Disease and bioanalytical assay methods were tested as covariates in the initial base model and appeared to be statistically significant for CL_1_ and k_des_, and therefore were included in the base model. Furthermore, 2 different residual variability components (one for NHL and one for ALL) were accounted to help characterize InO PK and to improve model stability before covariate selection. The impact of InO concentration data that were < LLOQ on the PK of InO was assessed using 2 modeling approaches, method 1 (M1) and method 3 (M3). The M1 approach omitted concentrations < LLOQ, and M3 maximized the likelihood for data ≥ LLOQ and treated PK data < LLOQ as censored [17]. The model fit from M3 was compared with the model obtained from M1 to determine if inclusion of data < LLOQ had a meaningful effect on the model parameters, particularly on InO clearance. The first-order conditional estimation method with interaction was implemented for the M1 analysis and the Laplacian approximation method for the M3 analysis.

### Covariate model development

Covariate relations were identified using generalized additive modeling (GAM) [18] on base model parameters and then tested in a stepwise manner with statistical criteria of *P *< 0.05 for the forward inclusion step and *P *< 0.001 for the backward elimination step. Baseline continuous covariates tested in the model included body surface area (BBSA), age, leukemic blasts in peripheral blood, creatinine clearance, alanine aminotransferase, aspartate aminotransferase, and total bilirubin (BBIL). Categorical covariates included sex, race, salvage line, concomitant medications (e.g., p-glycoprotein inhibitors, granulocyte colony-stimulating factors, hydroxyurea, and prior radiotherapy), concomitant rituximab use, disease type (B-cell ALL vs B-cell NHL and/or bioanalytical method), and hepatic impairment (assessed using the National Cancer Institute Organ Dysfunction Working Group criteria for hepatic impairment [19]).

### Model evaluation

Pharmacokinetic models were evaluated using change in objective function value (ΔOFV), graphically using goodness-of-fit plots, using 95% CIs around parameter estimates from nonparametric bootstrapping (*N* = 1000), and decreases in both interindividual and residual variability. A ΔOFV of 10.83 corresponded to a *P*-value of 0.001. The performance of the final model was evaluated by simulation data using final parameter estimates and conducting prediction- and variability-corrected visual predictive checks (pvcVPC [20]). Using patients’ characteristics, dosing, and sampling history, simulations were performed and concentration–time data were summarized using median, low, and high percentiles. Disease, bioanalytical assay methods, or both, were used to stratify simulated and observed data, and the concordance between individual observations and simulated values were assessed. In VPCs, all data < LLOQ were retained, and the 5^th^ and 95^th^ percentiles for observed data were calculated for those percentiles where data < LLOQ constituted a smaller fraction than the percentile in question.

### Model-based simulations

The final PK model was used to simulate the expected InO concentration–time course for the dosing regimen used in patients with ALL at a fixed dosing regimen of 1.8 mg/m^2^/cycle of InO (the maximum tolerated dose in NHL studies), administered at fractionated doses of 0.8 mg/m^2^ on day 1 and 0.5 mg/m^2^ on days 8 and 15 for cycle 1, lasting 21 days, and then up to 6 continuous cycles of 28 days (excluding any dose interruptions). The simulations were used to evaluate the effects of covariates, determine the spread of concentrations and approach to steady-state, and to compute steady-state PK parameters, such as area under the curve within a dosing interval (AUC_tau_), cumulative AUC, and half-life.

The NONMEM 7 level 2.0 and 3.0 was used for all model estimations, including stepwise covariate evaluation. Perl-speaks-NONMEM version 4.2.0 was used for nonparametric bootstrap and pvcVPCs, and SPLUS 8.0 and R version 2.15.2 was used for postprocessing and plotting. The NONMEM model code is shown in Online Resource 3.

## Results

### Patient characteristics and observed data

The data set for the analysis (cutoff date: March 8, 2016) comprised 8361 serum PK samples from 765 patients treated with InO (Online Resource 1); 2978 samples were contributed from patients with ALL, and 6272 samples were from patients with NHL.

The summaries of baseline categorical and continuous covariates are summarized in Online Resource 4 and Table 1. In the total analysis population, 60% of patients were men, 70% of patients were white, the median (range) baseline age was 61 years old (18–92 years), and the median baseline body surface area (BSA) was 1.84 m^2^ (1.13–2.81 m^2^). Patients with ALL had higher median baseline creatinine clearance (122 vs 82 mL/min) and alanine aminotransferase levels (33 vs 20 U/L) compared with patients with NHL. In the ALL population, 38% of patients (27 of 72 patients) were salvage 3 or greater in study 2 (study 1010), and all the patients in study 1 (study 1022) were salvage 1 or 2 (160 of 162 patients, with 2 patients’ statuses unknown). Compared with patients in study 1, patients in study 2 had higher median baseline absolute blast counts in the peripheral blood (1 × 10^9^ counts vs 0.4 × 10^9^ counts), absolute CD22+ blast counts in peripheral blood (0.9 × 10^9^ counts vs 0.3 × 10^9^ counts), and percentage of blasts in peripheral blood (12% vs 2%).

**Table 1:** Summary of baseline continuous covariates by disease

Covariate, median (range)	ALL overall (n = 234)	NHL overall (n = 531)	Total (N = 765)
Age, years	46.0 (20.0–79.0)	65.0 (18.0–92.0)	61.0 (18.0–92.0)
Body weight, kg	74.0 (30.9–154)	73.2 (33.5–148)	73.3 (30.9–154)
Body surface area, m^2^	1.86 (1.27–2.81)	1.83 (1.13–2.56)	1.84 (1.13–2.81)
Creatinine clearance, mL/min	122 (29.4–368)	81.8 (18.2–264)	93.1 (18.2–368)
Albumin, g/dL	3.80 (1.80–4.93)	4.00 (2.20–5.20)	3.92 (1.80–5.20)
Aspartate aminotransferase, U/L	26.0 (5.00–187)	24.5 (7.00–163)	25.0 (5.00-187)
Alanine aminotransferase, U/L	33.0 (5.00–161)	20.4 (3.00–236)	22.0 (3.00–236)
Total bilirubin, mg/dL	0.500 (0.100–2.16)	0.499 (0.100–3.90)	0.499 (0.100–3.90)
Absolute blast counts in peripheral blood, × 10^9^	0.823 (0.00–254)	NA	0.823 (0.00–254)
Absolute CD22+ blast counts in peripheral blood, × 10^9^	0.442 (0.00–252)	NA	0.442 (0.00–252)
Blasts in peripheral blood, %	4.00 (0.00–100)	NA	4.00 (0.00–100)
Blasts that are CD22+ in peripheral blood, %	98.7 (11.4–100)	NA	98.7 (11.4–100)

Summary statistics were calculated prior to any covariate imputations

*NA* not applicable

### Base pharmacokinetic model development

A 2-compartment linear clearance model excluding data < LLOQ (M1) reduced the OFV compared with a 1-compartment model. The 2-compartment linear clearance model was then compared with a time-dependent clearance model, which improved the fit of the data and reduced the OFV by 983 points. The time-dependent model was further improved by removing the random effect on peripheral compartment parameters (intercompartment clearance and volume of distribution in peripheral compartment) and including 2 separate proportional residual errors to account for disease, bioanalytical assay methods, or both (i.e., NHL [ELISA] and ALL [HPLC/MS/MS]). The effects of disease, the bioanalytical assay method, or both, were then tested on the PK parameters (CL_1_, CL_2_, and k_des_) and showed that patients with ALL had lower CL_1_ and k_des_ compared with patients with NHL. Correlation between the random effects in the variance–covariance matrix (OMEGA) diagonal were also evaluated with different OMEGA structures, resulting in a full OMEGA block (3) for CL_1_, volume of distribution in central compartment (V_1_), and CL_2_ and diagonal for k_des_. Using the M3 base model, data < LLOQ were included into the dataset; the parameter estimates differed by more than 15% compared with the M1 base model (data < LLOQ omitted). Furthermore, the η-shrinkage estimates of the M3 base model for CL_1_ and CL_2_ improved compared with the M1 base model **(**19% vs 15% and 31% vs 20%; Online Resource 5). Visual predictive checks showed misspecification in the terminal elimination phase when data < LLOQ were excluded; in contrast, including data < LLOQ showed improvement in the fit of the data (Online Resource 6).

### Final model results

The equations used to describe the final model estimation of typical values of CL_1_, CL_2_, V_1_, and k_des_ before interindividual variability were:

$${\text{CL}}_{ 1} = 0. 1 1 3 {\text{ L}}/{\text{h}} \cdot \left( { 1{-}0. 7 4 5\cdot {\text{PTST}}_{\text{ALL}} } \right) \cdot ({\text{BBSA}}/ 1. 8 4\;{\text{m}}^{ 2} )^{ 1. 5 4} \cdot( 1+ 0. 1 5 5\cdot {\text{RITX}})$$

$${\text{CL}}_{ 2} = 0. 3 6 9 {\text{ L}}/{\text{h}} \cdot ({\text{BBSA}}/ 1. 8 4\;{\text{m}}^{ 2} )^{ 1. 6 4}$$

$${\text{V}}_{ 1} = 6. 70{\text{ L}}/{\text{h}} \cdot ( 1+ 0. 7 7 4\cdot [{\text{BBSA}}{-} 1. 8 4\;{\text{m}}^{ 2} ])$$

$${\text{k}}_{\text{des}} = 0.0 3 3 7 {\text{h}}^{ - 1} \cdot \left( { 1{-}0. 8 60 \cdot {\text{PTST}}_{\text{ALL}} } \right) \cdot \left( {{\text{BLSTPB}}/ 5. 2 5\% } \right)^{ - 0.0 40 1}$$

where “RITX” (without rituximab) and “PTST_ALL_” (acute lymphoblastic leukemia patients and/or HPLC/MS/MS method) are 1 if applicable to each patient and otherwise 0.

For patients with ALL, the equations used to describe the typical values of CL_1_, CL_2_, V_1_, and k_des_ before interindividual variability were:

$${\text{CL}}_{ 1} = 0.0 3 3 3 {\text{ L}}/{\text{h}} \cdot ({\text{BBSA}}/ 1. 8 4\;{\text{m}}^{ 2} )^{ 1. 5 4}$$

$${\text{CL}}_{ 2} = 0. 3 6 9 {\text{ L}}/{\text{h}} \cdot ({\text{BBSA}}/ 1. 8 4\;{\text{m}}^{ 2} )^{ 1. 6 4}$$

$${\text{V}}_{ 1} = 6. 70{\text{ L}}/{\text{h}} \cdot ( 1+ 0. 7 7 4\cdot [{\text{BBSA}}{-} 1. 8 4\;{\text{m}}^{ 2} ])$$

$${\text{k}}_{\text{des}} = 0.00 4 7 2 {\text{h}}^{ - 1} \cdot \left( {{\text{BLSTPB}}/ 5. 2 5\% } \right)^{ - 0.0 40 1}$$

The total CL (i.e., CL = CL_1_ + CL_t_, where CL_t_ = CL_2 ·_ e ^[−kdes · Time]^) for patients with ALL without the covariate effects of BBSA and baseline percentage of blasts in peripheral blood (BSLTPB) on Cycle 4 Day 1 (Day 78 after first dose) was estimated to be 0.0333 L/h and calculated as:

$${\text{CL}} = 0.0 3 3 3 {\text{ L}}/{\text{h}} + \left( {0. 3 6 9 {\text{ L}}/{\text{h}} \cdot {\text{e}}^{{[ - 0.00 4 7 2\cdot 1 8 4 8\;{\text{h}}]}} } \right)$$

### Covariate model development

All potential covariates were tested in the M3 base model with the exception of sex because it was highly correlated with BBSA. Including BBSA as a covariate also corrected any previous trends associated with body weight (Online Resource 7). The variables baseline leukemia blasts expressing CD22 in peripheral blood and percentage of blasts in peripheral blood were only tested in patients with ALL because this information was not collected in patients with NHL. After screening of covariates with GAM, the following covariates were selected for statistical significance in stepwise covariate modeling: concomitant administration of rituximab with InO, hydroxyurea treatment, BLSTPB, baseline percentage of leukemic blasts expressing CD22 in peripheral blood, BBSA, and baseline albumin (BALB) on CL_1_ and CL_2_; BBSA on V_1_; salvage therapy, BBSA, and BLSTPB on k_des_. Age, race, and baseline creatinine clearance did not have a significant effect on InO PK parameters. After covariates were analyzed following step forward addition at a significance level of 0.05 and backward elimination approaches at a significance level of 0.001, with the likelihood ratio test assessing the significance of a covariate or covariates, the covariate effects retained in the final model were BBSA on CL_1_, CL_2_, and V_1_; absence of concomitant rituximab with InO on CL_1_; and BLSTPB on k_des_. A summary of the parameter estimates of the final covariate model is shown in Table 2.

**Table 2:** Parameter estimates of the final model

Parameter	Definition	NONMEM results OFV = 1450.357				Nonparametric bootstrap results		
		Estimate^c^	95% CI^a^		Shrinkage	Estimate	95% CI^b^	
			Lower	Upper	%	Median	Lower	Upper
CL_1_, L/h	Linear clearance	0.113	0.106	0.120	–	0.114	0.104	0.124
ALL effect^d^	/	− 0.745	− 0.778	− 0.712	–	− 0.747	− 0.777	− 0.712
BBSA effect	/	1.54	1.20	1.87	–	1.52	1.18	1.88
RITX + InO effect	/	0.155	0.0464	0.264	–	0.152	0.0565	0.266
CL_2_, L/h	Clearance associated with time-dependent clearance	0.369	0.332	0.406	–	0.373	0.338	0.420
BBSA effect	/	1.64	1.08	2.20	–	1.66	1.08	2.17
V_1_, L	Volume of distribution in central compartment	6.70	6.40	7.00	–	6.69	6.43	6.96
BBSA effect	/	0.774	0.620	0.928	–	0.774	0.657	0.887
k_des_, h^−1^	Decay coefficient of the time-dependent clearance	0.0337	0.0251	0.0423	–	0.0336	0.0249	0.0447
ALL effect^d^	/	− 0.860	− 0.897	− 0.823	–	− 0.859	− 0.893	− 0.806
BLSTPB effect	/	− 0.0401	− 0.0545	− 0.0257	–	− 0.0392	− 0.0589	− 0.0230
Q, L/h	Intercompartment clearance	0.0405	0.0349	0.0461	–	0.0418	0.0355	0.0507
V_2_, L	Volume of distribution in peripheral compartment	5.10	4.60	5.60	–	4.88	3.02	7.62
CL_1_ ω^2^	Variance–covariance matrix of the interindividual effects in CL_1_	42.3	0.147	0.211	18.9	0.170 (41.2)	0.113	0.258
CL_2_ ω^2^	Variance–covariance matrix of the interindividual effects in CL_2_	67.2	0.370	0.533	23.2	0.443 (66.6)	0.281	0.783
V_1_ ω^2^	Variance–covariance matrix of the interindividual effects in V_1_	41.2	0.150	0.190	15.8	0.169 (41.1)	0.129	0.209
k_des_ ω^2^	Variance–covariance matrix of the interindividual effects in k_des_	45.5	0.137	0.277	57.1	0.197 (44.4)	0.114	0.347
CL_1_–V_1_ ω^2^ (covariance)	/	0.156^e^	0.133	0.179	–	0.151	0.112	0.203
CL_1_–CL_2_ ω^2^ (covariance)	/	0.213^e^	0.175	0.251	–	0.208	0.136	0.298
CL_2_–V_1_ ω^2^ (covariance)	/	0.222^e^	0.191	0.253	–	0.218	0.160	0.291
${\text{O'}}^{2}_{\text{prop}} - {\text{NHL}}^{\text{f}}$	Variance of the NHL population	0.453	0.447	0.459	18.5	0.450	0.402	0.501
${\text{O'}}^{2}_{\text{prop}} - {\text{ALL}}^{\text{f}}$	Variance of the ALL population	0.619	0.612	0.626		0.611	0.555	0.684

%CV = percent coefficient of variation; RITX + InO = inotuzumab ozogamicin administered with rituximab

^a^The 95% CI was manually calculated using the following equation: Estimate ± 1.96 × SE. Standard error (SE) was obtained from the covariance step using the S Matrix in NONMEM (R and R/S Matrix had unsuccessful covariance steps); ^b^773 of 1000 bootstraps had successful minimization; ^c^interindividual variability of parameter estimates has been reported on the %CV scale, i.e. sqrt (ω^2^) as the parameters follow a log-normal distribution; ^d^ALL effect on CL_1_ and k_des_ accounted for different diseases (i.e., ALL vs NHL) and/or bioanalytical assay method (i.e., high-performance liquid chromatography with tandem mass spectrometry for ALL studies and ELISA for NHL studies); ^e^correlations were estimated to be 89.4% for CL_1_ − V_1_, 75.0% for CL_1_ − CL_2_, and 80.2% for CL_2_ − V_1_; ^f^2 residual errors were included in the model for different diseases (i.e., ALL vs NHL) and/or bioanalytical assay method (i.e., high-performance liquid chromatography with tandem mass spectrometry for ALL studies and ELISA for NHL studies)

The typical PK parameter estimates were 0.0113 L/h with 42.3% interindividual variability for CL_1_, 0.369 L/h with 67.2% interindividual variability for CL_2_, 0.0337 h^−1^ with 45.5% interindividual variability for k_des_, and 6.70 L with 41.2% interindividual variability for V_1_.

Patients with B-cell ALL had lower CL_1_ and k_des_ compared with patients with B-cell NHL. For patients with B-cell ALL, CL_1_ decreased by 75% (95% CI 71–78%) and k_des_ decreased by 86% (95% CI 82–90%) relative to patients with B-cell NHL. This effect could have been due to the disease type, differences in bioanalytical methodology, or both.

**Fig. 2:**
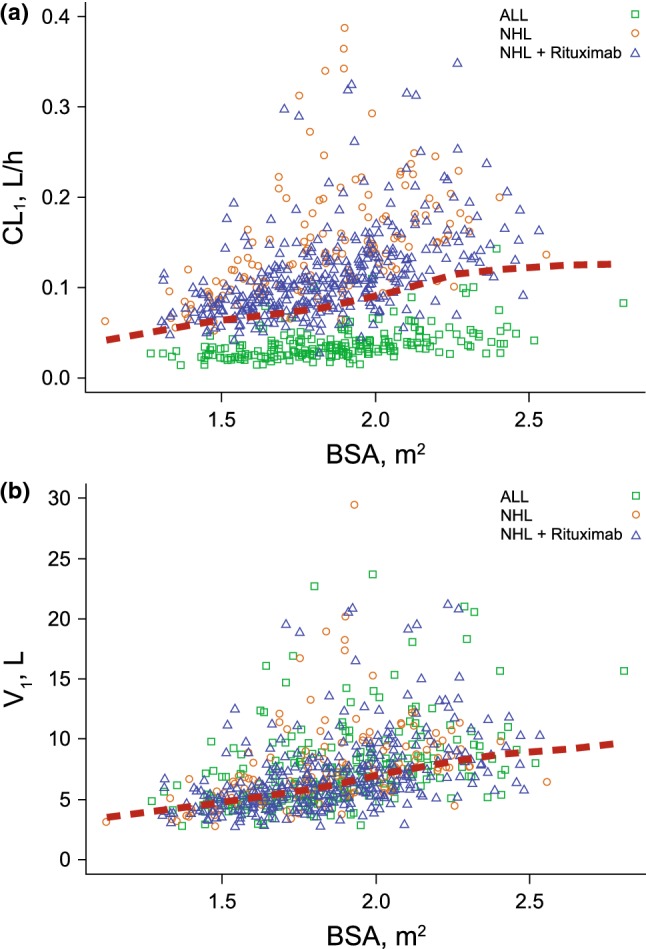
**a** Linear clearance^a^ or **b** Volume of distribution^b^ in central compartment versus baseline body surface area by patient type and those receiving rituximab. The red dotted lines are the locally weighted scatterplot smoothing trend line (LOESS). ^a^3 outliers of CL_1_ > 0.4 L/h were omitted from the plot for better visualization. ^b^1 outlier of V_1_ > 30 L was omitted from the plot for better visualization

**Fig. 3:**
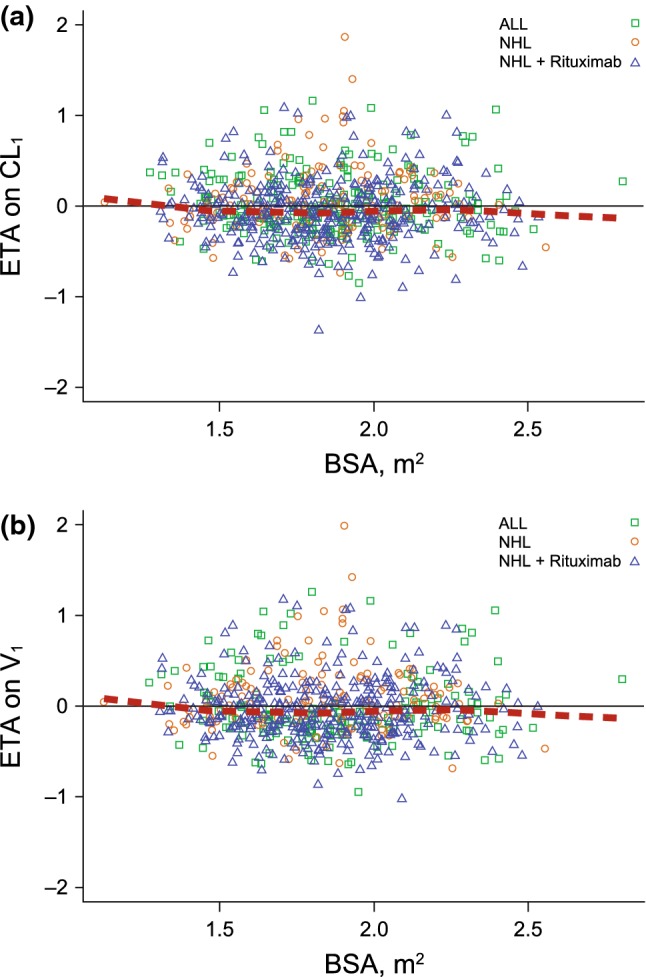
Final model empirical Bayes estimate of the interindividual random effect in **a** linear clearance or **b** volume of distribution in central compartment versus baseline body surface area by patient type and those receiving rituximab. The black solid lines are the reference line (y = 0) and the red dotted lines are the LOESS

An increase in BBSA was correlated with an increase in CL_1_, CL_2_, and V_1_ (Fig. 2). For individuals with a low BBSA of 1.55 m^2^ (10th percentile), CL_1_ decreased by 23%, CL_2_ by 25%, and V_1_ by 23%, resulting in higher exposures. Conversely, for individuals with a high BBSA of 2.21 m^2^ (90th percentile), CL_1_ increased by 33%, CL_2_ by 35%, and V_1_ by 29%, resulting in lower exposures. The empirical Bayes estimate of the interindividual random effects in a PK parameter (ETAs) on CL_1_, CL_2_, and V_1_ were centered on 0, demonstrating that the model adequately accounts for the BBSA effect (Fig. 3 and Online Resource 8).

**Fig. 4:**
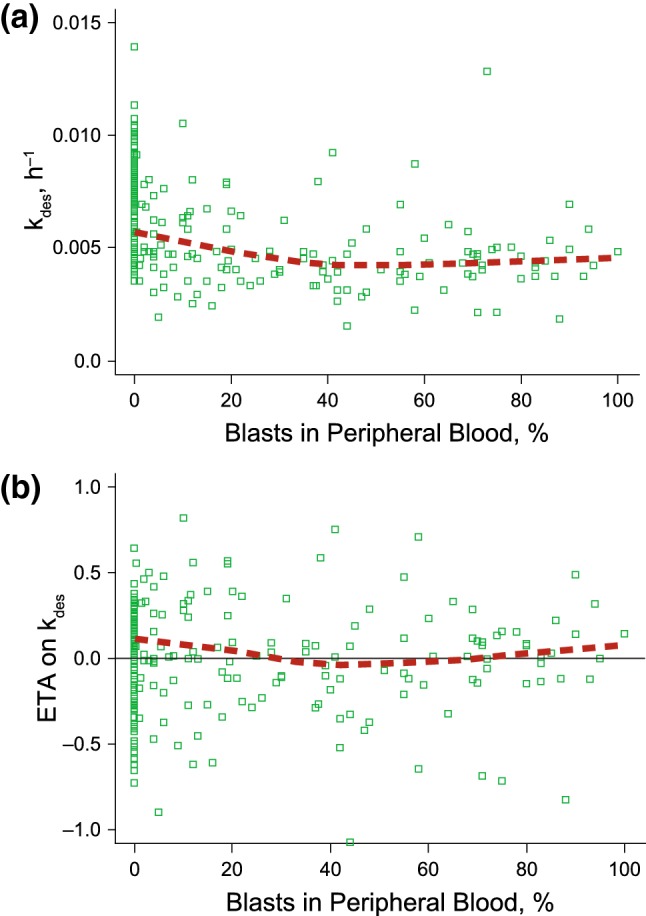
**a** Decay coefficient of the time-dependent clearance versus blasts in peripheral blood and **b** final model empirical Bayes estimate of the interindividual random effect in decay coefficient of the time-dependent clearance versus blasts in peripheral blood. The black solid line is the reference line (y = 0) and the red dotted lines are the LOESS

Increasing BLSTPB was associated with a decrease in k_des_, and therefore a decrease in the rate of decline in CL_t_ (Fig. 4). Relative to the typical k_des_ value (0.0333 h^−1^) at BLSTPB of 5% (50th percentile), k_des_ increased by 55% at BLSTPB of 0.0001% (10th percentile) and decreased by 10% at BLSTPB of 69% (90th percentile). For patients with B-cell ALL, after < 1 week on treatment (i.e., approximately 147 h, calculated as natural logarithm (ln) (2)/k_des_ when BLSTPB is 5%), the contribution of CL_t_ was reduced by 50%. The estimated 55% increase and 10% decrease in k_des_ led to variations in the time corresponding to a 50% reduction in CL_t_, which ranged from 95 to 163 h. For the 10th percentile (BLSTPB of 0.0001%), 50th (5%), and 90th (69%) of BLSTPB, this contribution of CL_t_ on total clearance became negligible after 5 half-lives transpired, or by 2.8 weeks (for the 10th percentile), 4.4 weeks (for the 50th percentile), and 4.8 weeks (for the 90th percentile). The model ETAs on k_des_ versus BLSTPB were centered near 0, demonstrating that the final model adequately accounted for fluctuations in the BLSTPB. The absence of concomitant rituximab use resulted in an estimated increase of CL_1_ by 16% (95% CI 5–26%) in patients receiving single-agent InO versus those receiving rituximab plus InO. The model ETAs on concomitant rituximab use were centered near 0, showing that the final model adequately accounted for the effects of concomitant rituximab use (Online Resource 9).

**Fig. 5:**
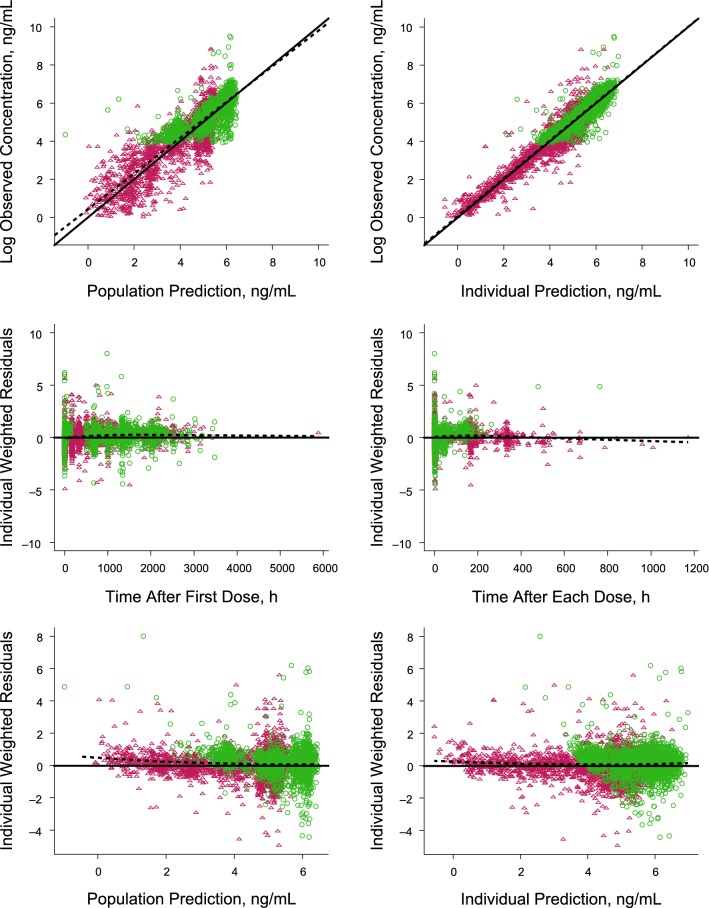
Goodness-of-fit diagnostic plots. In the plots of observed versus predicted, green circles (NHL) and maroon triangles (ALL) are individual data; solid line and dotted line show the reference line (y = x) and linear regression line based on the individual data. In the scatter plots of residuals, circle (NHL) and triangle (ALL) points are individual data; solid lines are the reference line (y = 0) and dotted lines are the locally weighted scatterplot smoothing trend line. Observed data, individual predictions, and population prediction values are log transformed. Data are represented on the natural logarithmic scale

**Fig. 6:**
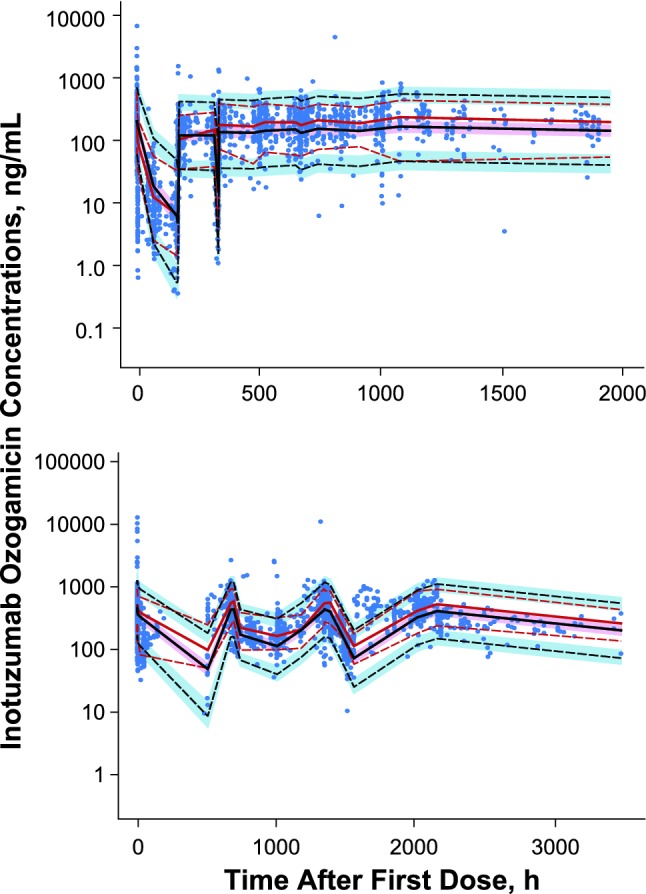
Prediction- and variability-corrected visual predictive check in **a** patients with ALL and **b** patients with NHL. The red lines show median (solid) and the 5th and 95th percentiles (dash) of the observed concentrations. The black lines show median (solid) and the 5th and 95th percentiles (dash) of the simulated concentrations, and shaded regions show the 95% CIs on quantities obtained by simulations (N = 1000)

**Fig. 7:**
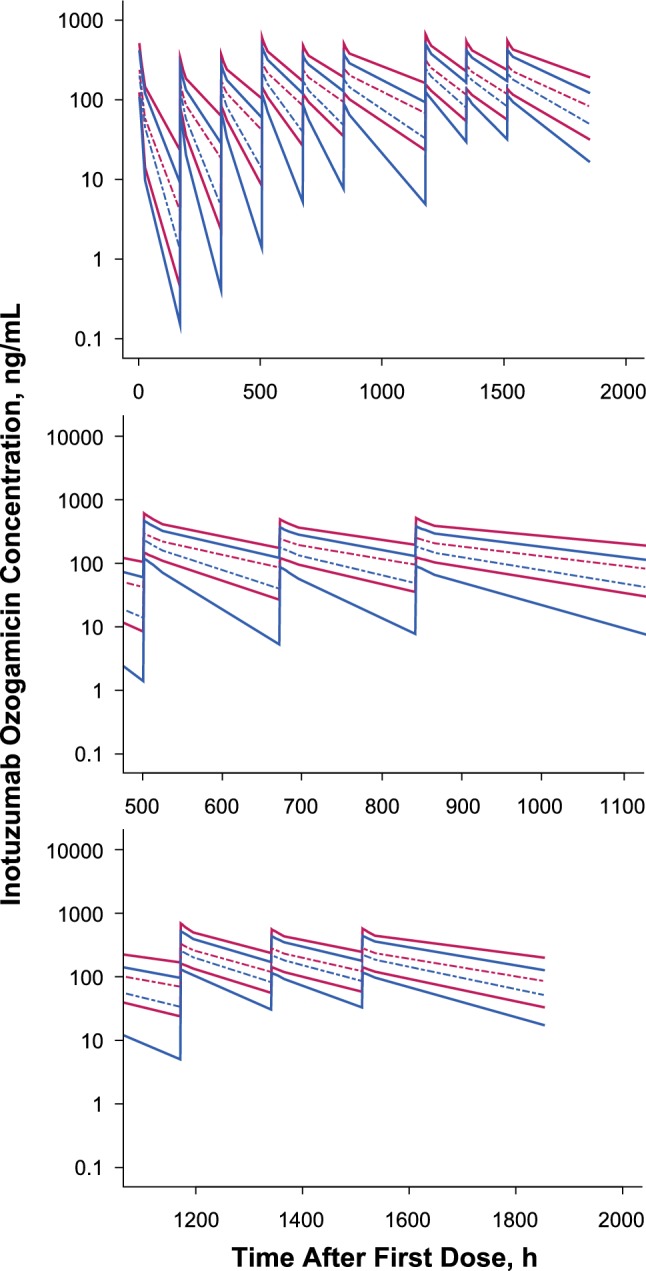
Simulations of effects of covariates on inotuzumab ozogamicin concentrations in ALL patients for **a** 1.8 mg/m^2^ for 3 Cycles, **b** Cycle 2, and (C) Cycle 3. Red lines denote highest InO exposure (low BBSA/low BLSTPB) and blue lines denote lowest InO exposure (high BBSA/high BLSTPB). Medians are shown with dashed lines, and the 5th and 95th percentiles are shown with solid lines

**Fig. 8:**
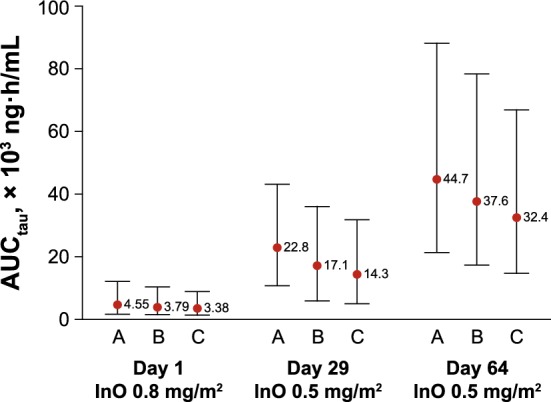
Simulation of covariate effects on inotuzumab ozogamicin AUC_tau_. Red circles are the 50th percentile (the numbers next to the red circles represent medians) and black bars represent the 5th and 95th percentiles of the simulated data (N = 1000). Tau is 7 days for days 1 and 29 and 14 days for day 64. A = low BBSA (1.55 m^2^) and low BLSTPB (0.0001%); B = median BBSA (1.84 m^2^) and median BLSTPB (5.25%); C = high BBSA (2.21 m^2^) and high BLSTPB (69%). tau = dosing interval

The goodness-of-fit diagnostic plots of the covariate model did not indicate any model deficiencies (Fig. 5). The dependencies of the random effects on covariates did not show any further trends unaccounted for by the model. Visual predictive check simulations indicated good agreement between observed and simulated data for patients with ALL and NHL (Fig. 6) and covariates (Figs. 7 and 8).

Shrinkage was small (< 30%) for all parameters except the k_des_ (57%; Table 2). k_des_ is one of the components of the total clearance parameter (i.e., CL_t_ = CL2 · e ^[−kdes · Time])^ and its contribution changes over time. Hence, for patients with ALL, after approximately 147 h (i.e., ln (2)/k_des_ when BLSTPB is 5.25%), and < 1 week on treatment, the contribution of CL_t_ is reduced by 50%. Thus, the actual high shrinkage on k_des_ does not significantly affect the individual clearance estimates.

### Model-based simulations

The combination covariate effects of BBSA and BLSTPB on the typical concentration–time courses and InO exposures for patients with B-cell ALL receiving a fixed-dose regimen of 1.8 mg/m^2^/cycle, administered at fractionated doses of 0.8 mg/m^2^ on day 1 and 0.5 mg/m^2^ on days 8 and 15 for the first 21 days and then every 28 days for 3 continuous cycles, are shown in Figs. 7 and 8. Patients with the highest exposure (i.e., 10th percentile for BBSA and BLSTPB) and lowest exposure (i.e., 90th percentile for BBSA and BLSTPB) showed substantial overlap in the simulated InO concentrations and exposure. Model-based simulation of InO concentration–time curves showed that steady-state levels were achieved after 3 cycles of treatment following multiple dosing with InO of 1.8 mg/m^2^/cycle in patients with ALL. The predicted median AUC_tau_ on day 1 of cycle 4 (C4D1) was 29,800 ng·h/mL, terminal beta half-life was 293 h (12 days), and geometric mean ratio for InO accumulation, calculated as the ratio of AUC_tau_ on C4D1 over AUC_inf_ was 5.9 (95% CI 5.67–6.15).

## Discussion

In an effort to fully characterize the PK properties of InO, a population PK analysis was conducted by pooling sparse and dense PK data from adult patients with relapsed or refractory ALL and NHL. By using this population PK approach, integrated information and a more robust structural PK model was obtained to describe InO PK. This analysis demonstrated that a 2-compartment PK model with a linear and time-dependent clearance component accurately describes the concentration–time course of InO in patients with R/R B-cell ALL and NHL. The PK model for InO is consistent with previous analyses for similar types of molecules; time-dependent PK is often demonstrated in antibodies that target B-cell receptors (e.g., rituximab disposition was described using a time-dependent clearance model [CL_t_] [16]). In contrast, target-mediated drugs demonstrate nonlinear drug disposition, which is probably due to the depletion or changes in target or target expression levels over time, as opposed to target-mediated elimination saturation [15].

Generally, the population PK of InO was similar to the population PK seen with other therapeutic monoclonal antibodies [21]. In patients with ALL, the CL_1_ and k_des_ of InO was 75 and 86% lower than in patients with NHL. Because each bioanalytical method was used exclusively for measurement of InO PK in a particular tumor type in the population PK analysis, the effect of bioanalytical assay method included any potential differences in tumor type (i.e., relapsed or refractory ALL versus relapsed or refractory NHL). NHL is a malignant solid tumor of the immune system whereas ALL is a cancer of the blood and bone. These different tumor types may have different binding receptors and CD22 antigen expression.

An increase in BBSA was correlated with an increase in CL_1_, CL_2_, and V_1_. Because InO dosing therapy is calculated as a function of BSA, the influence of BBSA on InO disposition supports the current dosing regimen based on an individual patient’s BSA.

In patients with ALL, high values of BLSTPB were correlated with a decrease in k_des_. Increasing BLSTPB was associated with a decrease in k_des_, and therefore, a decrease in the rate of decline in the time-dependent component of the total clearance, C_t_, with time. Because leukemic blast cells (a measure of tumor burden) in patients with ALL tend to be localized to the bone marrow and/or circulating blood, these blasts are likely to be more rapidly exposed to InO, resulting in faster clearance of the drug compared with in patients with solid tumors. It is important to mention that CL_t_ (i.e., CL_t_ = CL_2_ · e ^[−kdes · Time]^) is one of the components of the total clearance parameter and its contribution changes over time. For patients with ALL, after < 1 week on treatment, the contribution of CL_t_ is reduced by 50%. Thus, the actual impact of any covariate on k_des_ does not translate into a similar magnitude of change in elimination rate. Given the magnitude of estimated change from the typical value of k_des_ and given that CL_2_ is not the only clearance component, BLSTPB is not considered to have a significant effect on InO disposition over the treatment duration.

In studies with ALL patients, InO was administered as a single-agent, and in the studies with NHL patients, InO was administered either as a single-agent, in combination with rituximab, or in combination with rituximab plus chemotherapy. In order to account for any concomitant rituximab effects on InO PK, rituximab was evaluated as a covariate in the model. In the population PK analysis, the absence of concomitant rituximab effects on CL_1_ resulted in an estimated 16% increase in CL_1_ (95% CI 5–26%) relative to patients who were given rituximab (in NHL studies only). However, this increase in InO CL_1_ was less than the corresponding estimated interindividual variability (42%) in inotuzumab ozogamicin CL_1_. Therefore, the effect of rituximab on the PK of inotuzumab ozogamicin was not considered to be clinically relevant. It should be noted that inotuzumab ozogamicin is indicated as a single-agent therapy, and not in combination with rituximab, in adult patients with relapsed or refractory ALL.

Additionally, other demographic factors (e.g., age, race, and sex) and measures of renal and hepatic function were evaluated and found not to affect the PK of InO. Together, these findings suggest that no dose adjustment is needed for patients with ALL receiving InO treatment.

## Electronic supplementary material

Below is the link to the electronic supplementary material.

Supplementary material 1 (DOCX 363 kb)
