# Supplementary material for: Population pharmacokinetics of inotuzumab ozogamicin in relapsed/refractory acute lymphoblastic leukemia and non-Hodgkin lymphoma
Source: J Pharmacokinet Pharmacodyn. 2019 Mar 11;46(3):211–22. doi: 10.1007/s10928-018-9614-9 (PMC6529376; doi:10.1007/s10928-018-9614-9)
Supplement: Supplementary file 1 — Supplementary material 1 (DOCX 363 kb) [file 10928_2018_9614_MOESM1_ESM.docx]

**Supplementary Materials**

**Article title:** Population Pharmacokinetics of Inotuzumab Ozogamicin in Relapsed/Refractory Acute Lymphoblastic Leukemia and Non-Hodgkin Lymphoma

**Journal name:** *Journal of Pharmacokinetics and Pharmacodynamics*

**Authors:** May Garrett,^1^ Ana Ruiz-Garcia,^1^ Kourosh Parivar,^1^ Brian Hee,^1^ Joseph Boni^2^

**Affiliations:** ^1^Pfizer Oncology, San Diego, CA, USA: ^2^Pfizer Oncology, Collegeville, PA, USA

**Email of corresponding author:** May.Garrett@pfizer.com

**Online Resource 1. Clinical Studies Included in Population PK Analysis.**

| Study | Design | InO Dosing | Total Number of Patients Included in PK Analysis | PK  Sampling^a^ |
| --- | --- | --- | --- | --- |
| **Patients With B-Cell ALL Who Received Single-Agent InO** | | | | |
| Study 1  (Study 1022; clinicaltrials.gov: NCT01564784) | Phase 3,  global,  multicenter,  2‑arm,  randomized | Arm A (n=164 randomized [164 treated]):  InO 1.8 mg/m^2^/21- to 28-day cycle (0.8, 0.5, and 0.5 mg/m^2^ on days 1, 8, and 15)  Arm B (n=162 randomized [143 treated]):  FLAG, cytarabine plus mitoxantrone, or  HIDAC (21- to 28-day cycle) | 162 | Sparse PK;  cycles 1, 2,  and 4 |
| Study 2  (Study 1010; clinicaltrials.gov: NCT01363297) | Phase 1/2,  open‑label,  single-arm,  dose‑escalation | Phase 1 dose finding:  1.2 mg/m^2^/28-day cycle (0.8 and 0.4 mg/m^2^  on days 1 and 15, n=3)  1.6 mg/m^2^/28-day cycle (0.8, 0.4, and  0.4 mg/m^2^ on days 1, 8, and 15, n=12)  1.8 mg/m^2^/28-day cycle (0.8, 0.5, and  0.5 mg/m^2^ on days 1, 8, and 15, n=9)  Phase 1 dose expansion:  1.8 mg/m^2^/28-day cycle (0.8, 0.5, and  0.5 mg/m^2^ on days 1, 8, and 15, n=13) | 72 | Sparse PK;  cycles 1, 2,  and 4 |
| **Patients With B-Cell NHL Who Received Single-Agent InO** | | | | |
| Study 3  (Study 1002; clinicaltrials.gov: NCT00073749) | Phase 1,  FIH,  open‑label,  multicenter,  dose‑escalation | Phase 1:  0.4 mg/m^2^ q3w (n=2)  0.8 mg/m^2^ q3w (n=5)  1.34 mg/m^2^ q3w (n=11)  1.8 mg/m^2^ q3w (n=6)  2.4 mg/m^2^ q3w (n=6)  Phase 2:  1.8 mg/m^2^ q4w (n=49) | 59 | Full PK;  cycles 1, 2, 3,  and 4 |
| Study 4 (Study 1016) | Phase 1,  dose‑escalation | 1.3 mg/m^2^ q4w (n=3)  1.8 mg/m^2^ q4w (n=10) | 13 | Full PK;  cycles 1, 2,  and 3;  Sparse PK;  cycle 4 |
| Study 5  (Study 1007; clinicaltrials.gov: NCT00868608) | Phase 2,  single-arm | 1.8 mg/m^2^ q4w | 75 | Sparse PK;  cycles 1, 3, 4,  and 6 |
| **Patients With B-Cell NHL Who Received InO in Combination With Rituximab** | | | | |
| Study 6  (Study 1005; clinicaltrials.gov: NCT00724971) | Phase 1,  open-label,  multicenter | InO  1.8 mg/m^2^ q4w  Rituximab  375 mg/m^2^ q4w | 10 | Full PK;  cycles 1, 2,  and 3 |
| Study 7  (Study 1004; clinicaltrials.gov: NCT00299494) | Phase 1/2,  open-label,  dose‑escalation | Part 1:  InO  0.8 mg/m^2^ (n=5)  1.3 mg/m^2^ (n=3)  1.8 mg/m^2^ (n=7)  plus rituximab 375 mg/m^2^ q4w  Part 2:  InO 0.8 mg/m^2^ plus  rituximab 375 mg/m^2^ q4w (n=104) | 118 | Full PK;  cycles 1, 2,  and 3 |
| Study 8  (Study 1001; clinicaltrials.gov: NCT00867087) | Phase 2,  open-label,  multicenter | InO  1.8 mg/m^2^ q3w  Rituximab  375 mg/m^2^ q3w | 53 | Sparse PK;  cycles 1, 2,  and 4 |
| Study 9  (Study 1006; clinicaltrials.gov: NCT00562965) | Phase 3,  open-label,  randomized 1:1,  active‑comparator | Arm 1 (n=15):  InO  1.8 mg/m^2^ q4w  Plus rituximab  375 mg/m^2^ q4w  Arm 2 (n=14):  R-CVP or R-FND | 14 | Sparse PK;  cycles 1, 2, 3,  and 4 |
| Study 10  (Study 1008; clinicaltrials.gov: NCT01232556) | Phase 3,  multicenter,  randomized 1:1,  active‑comparator | Arm 1 (n=166 randomized [164 treated]):  InO (1.8 mg/m^2^) on day 2  of a 4-week cycle, plus rituximab 375 mg/m^2^  on day 1 of a 4-week cycle.  Arm 2 (n=172 randomized [167 treated]):  Rituximab 375 mg/m^2^ on day 1 +  bendamustine 120 mg/m^2^ on days 1 and 2 of  a 4-week cycle or rituximab 375 mg/m^2^ on days 1, 8, 15, and 22 of cycle 1 and on day 1 for all other cycles, + gemcitabine 1000 mg/m^2^ on days 1, 8, and 15 of a 4-week cycle. | 145 | Sparse PK;  cycles 1, 3,  and 4 |
| **Patients With NHL Who Received InO in Combination With Rituximab Plus Chemotherapy** | | | | |
| Study 11  (Study 1003; clinicaltrials.gov: NCT01055496) | Phase 1,  open-label,  multicenter,  1-arm,  dose‑finding | Arm 1 (n=48):  InO (0.4, 0.8, 1.3 mg/m^2^)  + R‑CVP  Arm 2 (n=55):  InO (0.8 mg/m^2^) +  R‑GDP | 44 | Sparse PK;  cycles 1, 3,  and 4 |

FIH=first-in-human; FLAG=cytarabine+fludarabine+GCSF; FND=rituximab, fludarabine, mitoxantrone, and dexamethasone; HIDAC=high-dose cytarabine; R-CVP=rituximab, cyclophosphamide, vincristine, and prednisone; R-FND=rituximab, fludarabine, mitoxantrone, and dexamethasone; R-GDP=rituximab, gemcitabine, dexamethasone, and cisplatin.

^a^Sparse PK means that only a few blood samples were collected at each visit (eg, 1‒3 samples) and Full PK means that multiple blood samples were collected at each visit (eg, every hour over a 24-hour period).

**Online Resource 2. Specifications for Bioanalytical Assay of Inotuzumab Ozogamicin Concentrations.**

| **Study** | **Assay Method** | **Linear Range, ng/mL** | **Precision,**  **%CV^a^** | **Bias, %RE^a^** |
| --- | --- | --- | --- | --- |
| ALL study |  |  |  |  |
| 1 | HPLC/MS/MS | 1.00‒500 | ≤11.3 | ‒15.3 to 5.58 |
| 2 | HPLC/MS/MS | 1.00‒500 | ≤13.5 | ‒5.25 to 14.5 |
| NHL |  |  |  |  |
| 3 | ELISA | 418‒11,200^b^  52.2‒1400^c^ | ≤13.8  ≤14.3 | ‒10.2 to 1.1  ‒10.5 to ‒4.2 |
| 4 | ELISA | 52.2‒1400^c^ | ≤14.8 | ‒8.1 to 1.0 |
| 5 | ELISA | 50.0‒1400 | ≤8.86 | ‒2.91 to 0.150 |
| 6 | ELISA | 52.2‒1400 | ≤8.8 | ‒7.3 to 6.3 |
| 7 | ELISA | 52.2‒1400 | ≤12.4 | ‒4.6 to ‒4.2 |
| 8 | ELISA | 50.0‒1400 | ≤8.13 | ‒2.86 to 1.59 |
| 9 | ELISA | 50.0‒1400 | ≤7.53 | ‒4.83 to ‒2.11 |
| 10 | ELISA | 50.0‒1400 | ≤22.6 | 2.13 to 8.95 |
| 11 | ELISA | 50.0‒1400 | ≤7.68 | 0.317 to 9.81 |

%RE=percent relative error.

^a^Statistics (%RE and %CV) are based on mean assay performance of low, mid, high, and diluted (if applicable) quality control samples from all analytical batches meeting acceptance criteria; ^b^includes LLOQ 667 ng/mL; ^c^includes LLOQ 83.4 ng/mL.

**Online Resource 3. NONMEM Code for the Final Model.**

$PROBLEM run1.mod ; Supplement Analysis

$INPUT C PROT NSID ID XDATE=DROP XTIME=DROP TIME CYCL OCC

XMDV=DROP MDV XEVI=DROP EVID FLAG=DROP XDV=DROP XDV3=DROP XLND=DROP DV

RATE AMT DOSE AGE SEX RACE WT=DROP BWT BSA=DROP BBSA CCL=DROP BCCL

ALB=DROP BALB ALK=DROP BALK=DROP ALT=DROP BALT AST=DROP BAST BIL=DROP BBIL

RITX RITU COMB GCSF SALV HYDR ECOG=DROP BECOG RADIO CHPH=DROP PGP

BMB=DROP BBMB BLAS=DROP BHGRADE HGRADE=DROP XBLQ=DROP

BLSTABL CD22BLST BLSTPB CD22PPB LOQ PTST ASIA JAPA LBLSTABL LCD22BLST

$DATA B193_PPK_PMAR202_SUPPLEMENT_27JUN2016_tafd.csv WIDE IGNORE=C

$SUBROUTINE ADVAN6 TOL=6

$MODEL NCOMP=2 COMP=(CENTRAL) ; 1

COMP=(PHP) ; 2

$PK

;;; V1BBSA-DEFINITION START

V1BBSA = ( 1 + THETA(15)*(BBSA - 1.84))

;;; V1BBSA-DEFINITION END

;;; V1-RELATION START

V1COV=V1BBSA

;;; V1-RELATION END

;;; KDESBLSTPB-DEFINITION START

IF(BLSTPB.EQ.-99) THEN

KDESBLSTPB = 1

ELSE

KDESBLSTPB = ((BLSTPB/5.25)**THETA(14))

ENDIF

;;; KDESBLSTPB-DEFINITION END

;;; KDES-RELATION START

KDESCOV=KDESBLSTPB

;;; KDES-RELATION END

;;; CL2BBSA-DEFINITION START

CL2BBSA = ((BBSA/1.84)**THETA(13))

;;; CL2BBSA-DEFINITION END

;;; CL2-RELATION START

CL2COV=CL2BBSA

;;; CL2-RELATION END

;;; CL1RITX-DEFINITION START

IF(RITX.EQ.1) CL1RITX = 1 ; Most common

IF(RITX.EQ.0) CL1RITX = ( 1 + THETA(12))

;;; CL1RITX-DEFINITION END

;;; CL1BBSA-DEFINITION START

CL1BBSA = ((BBSA/1.84)**THETA(11))

;;; CL1BBSA-DEFINITION END

;;; CL1-RELATION START

CL1COV=CL1BBSA*CL1RITX

;;; CL1-RELATION END

IF (EVID.EQ.1.OR.EVID.EQ.4) THEN

TDOS=TIME

TAD=0.0

ENDIF

IF (EVID.NE.1.AND.EVID.NE.4) TAD=TIME-TDOS

;;

IF(PTST.EQ.1) CLPTST=1

IF(PTST.EQ.2) CLPTST=(1+THETA(9)) ; ALL on CL1

;;

IF(PTST.EQ.1) KDPTST=1

IF(PTST.EQ.2) KDPTST=(1+THETA(10)) ; ALL on KDES

TVCL1 = THETA(1) ; linear clearance

TVCL1 = CL1COV*TVCL1

TVV1 = THETA(2)

TVV1 = V1COV*TVV1

TVCL2 = THETA(3) ; clearance associated w/time-dependent clearance

TVCL2 = CL2COV*TVCL2

TVKDES = THETA(4) ; decay coefficient associated w/ time-dependent clearance

TVKDES = KDESCOV*TVKDES

TVQ = THETA(5)

TVV2 = THETA(6)

CL1 = TVCL1*CLPTST*EXP(ETA(1))

V1 = TVV1*EXP(ETA(2))

CL2 = TVCL2*EXP(ETA(3))

KDES = TVKDES*KDPTST*EXP(ETA(4))

Q = TVQ

V2 = TVV2

S1 = V1

K12 = Q/V1

K21 = Q/V2

$DES

C1 = A(1)/V1

CL= CL1 + CL2 * EXP(-KDES*T)

K10 = CL/V1

DADT(1) = -K12*A(1) + K21*A(2) - K10*A(1)

DADT(2) = -K21*A(2) + K12*A(1)

$ERROR

SIG=THETA(7) ; prop error for NHL

IF(PTST.EQ.2) SIG=THETA(8) ; prop error for ALL

LLOQ=LOG(LOQ) ; log-transformed because DV=log(CONC)

IPRED = LOG(0.0001)

IF(F.GT.0) IPRED = LOG(F)

DUM = (LLOQ-IPRED)/SIG

CUMD = PHI(DUM)

;-------------------------------------------------

BLQ = 0

IF(DV.LT.LLOQ) BLQ = 1

;-- Prediction DV>=LLOQ --------------------------

W = SIG ; residual error

IRES = DV-IPRED

IWRES = IRES/W

IF(DV.GE.LLOQ) THEN

F_FLAG = 0

Y = IPRED+W*ERR(1)

ENDIF

;-- Likelihood DV<LLOQ --------------------------

IF(DV.LT.LLOQ) THEN

F_FLAG = 1

Y = CUMD

ENDIF

;------------------------------------------------

$THETA (0,0.118248) ; 1 CL1

$THETA (0,6.63858) ; 2 V1

$THETA (0,0.367849) ; 3 CL2

$THETA (0,0.0321833) ; 4 KDES

$THETA (0,0.0398226) ; 5 Q

$THETA (0,5.46741) ; 6 V2

$THETA (0,0.45281) ; 7 prop.err for NHL

$THETA (0,0.640802) ; 8 prop.err for ALL

$THETA (-1,-0.7164) ; 9 ALL on CL1

$THETA (-1,-0.830125) ; 10 ALL on KDES

$THETA (-1000000,1.53,1000000) ; CL1BBSA1

$THETA (-1,0.154,5) ; CL1RITX1

$THETA (-1000000,1.63,1000000) ; CL2BBSA1

$THETA (-1000000,-0.0399,1000000) ; KDESBLSTPB1

$THETA (-1.030,0.771,1.388) ; V1BBSA1

$OMEGA BLOCK(3)

0.258271 ; 1.IIV.CL1

0.216144 ; 1-2.COV.CL1.V1

0.205707 ; 2.IIV.V1

0.326711 ; 1-3.COV.CL1.CL2

0.29147 ; 2-3.COV.CL2.V1

0.600566 ; 3.IIV.CL2

$OMEGA BLOCK(1)

0.254607 ; 4.IIV.KDES

$SIGMA 1 FIX

$ESTIMATION METHOD=COND INTER LAPLACIAN SLOW NUMERICAL SIGDIG=2

MAXEVAL=9990 NOABORT POSTHOC FILE=run1.ext MSFO=MSF20.msf

$COV UNCONDITIONAL PRINT=E MATRIX=S

$TABLE ID PROT PTST TIME TAD IPRED IWRES CWRES CWRESI CRESI CPREDI NPDE BLQ

MDV EVID DOSE CL CL1 CL2 V1 Q V2 KDES NOPRINT ONEHEADER FORMAT=sF14.4 FILE=sdtab1

$TABLE ID PROT PTST CL1 CL2 V1 Q V2 KDES ETA1 ETA2 ETA3 ETA4

NOPRINT NOAPPEND ONEHEADER FORMAT=sF14.4 FILE=patab1

$TABLE ID PROT PTST AGE BWT BBSA BCCL BALB BALT BAST BBIL BBMB BLSTABL

CD22BLST BLSTPB CD22PPB LBLSTABL LCD22BLST ETA1 ETA2 ETA3 ETA4 DOSE

NOPRINT NOAPPEND ONEHEADER FORMAT=sF14.4 FILE=cotab1

$TABLE ID PROT PTST SEX RACE ASIA JAPA BECOG PGP RITX RITU

BHGRADE COMB GCSF SALV HYDR RADIO ETA1 ETA2 ETA3 ETA4

NOPRINT NOAPPEND ONEHEADER FORMAT=sF14.4 FILE=catab1

$TABLE C PROT CYCL OCC NSID ID TIME TAD MDV EVID DV RATE AMT DOSE BBSA BLSTPB RITX

CL CL1 CL2 V1 Q V2 KDES NOPRINT NOAPPEND ONEHEADER FORMAT=sF14.4 FILE=simtab1

**Online Resource 4. Summary of Baseline Categorical Covariates by Disease.**

| Covariate, n (%) | Category | ALL Total  n=234 | NHL Total  n=531 | Total  N=765 |
| --- | --- | --- | --- | --- |
| Sex | Male | 141 (60.3) | 317 (59.7) | 458 (59.9) |
|  | Female | 93 (39.7 ) | 214 (40.3) | 307 (40.1) |
| Race | White | 165 (70.5) | 369 (69.5) | 534 (69.8) |
|  | Black | 6 (2.56) | 14 (2.64) | 20 (2.61) |
|  | Asian^a^ | 24 (10.3) | 30 (5.65) | 54 (7.06) |
|  | Japanese | 13 (5.56) | 88 (16.6) | 101 (13.2) |
|  | Other | 23 (9.83) | 30 (5.65) | 53 (6.93) |
|  | Unknown | 3 (1.28) | 0 (0.00) | 3 (0.392) |
| Baseline ECOG performance score | 0 | 74 (31.6) | 282 (53.1) | 356 (46.5) |
|  | 1 | 120 (51.3) | 199 (37.5) | 319 (41.7) |
|  | 2 | 37 (15.8) | 37 (6.97) | 74 (9.67) |
|  | 3 | 3 (1.28) | 9 (1.69) | 12 (1.57) |
|  | Unknown | 0 | 4 (0.753) | 4 (0.523) |
| Baseline  NCI ODWG  criteria for  hepatic impairment | A (normal) | 167 (71.4) | 443 (83.4) | 610 (79.7) |
|  | B1 (mild) | 58 (24.8) | 75 (14.1) | 133 (17.4) |
|  | B2 (mild) | 8 (3.42) | 9 (1.69) | 17 (2.22) |
|  | C (moderate) | 1 (0.427) | 2 (0.377) | 3 (0.392) |
|  | D (severe) | 0 | 1 (0.188) | 1 (0.131) |
|  | Unknown | 0 | 1 (0.188) | 1 (0.131) |
| Prior radiotherapy | No | 177 (75.6) | 397 (74.8) | 574 (75.0) |
|  | Yes | 57 (24.4) | 134 (25.2) | 191 (25.0) |
| Salvage line | Salvage 1 | 128 (54.7) | NA | 128 (16.7) |
|  | Salvage 2 | 77 (32.9) | NA | 77 (10.1) |
|  | Salvage 3+ | 27 (11.5) | NA | 27 (3.53) |
|  | Unknown | 2 (0.855) | 531 (100) | 533 (69.7) |
| Combination therapy  with rituximab | No | 234 (100) | 147 (27.7) | 381 (49.8) |
|  | Yes | 0 | 384 (72.3) | 384 (50.2) |
| Combination treatment^b^ | No | 166 (70.9) | 157 (29.6) | 323 (42.2) |
|  | Yes | 68 (29.1) | 374 (70.4) | 442 (57.8) |
| Stimulatory treatment^c^ | No | 148 (63.2) | 434 (81.7) | 582 (76.1) |
|  | Yes | 86 (36.8) | 97 (18.3) | 183 (23.9) |
| Concomitant hydroxyurea | No | 221 (94.4) | 531 (100) | 752 (98.3) |
|  | Yes | 13 (5.56) | 0 (0.00) | 13 (1.70) |
| P-glycoprotein inhibitors^d^ | No | 187 (79.9) | 439 (82.7) | 626 (81.8) |
|  | Yes | 47 (20.1) | 92 (17.3) | 139 (18.2) |

ECOG=Eastern Cooperative Oncology Group; NCI ODWG=National Cancer Institute Organ Dysfunction Working Group.

^a^Asian and Japanese patients are presented separately in the table above. However, in the stepwise covariate modeling, the Asian covariate included Japanese patients when tested against non-Asian patients; ^b^intercurrent or concomitant hydroxyurea, corticosteroid, or rituximab if administered concurrently during the first 2 cycles of InO; ^c^stimulatory treatment with granulocyte colony-stimulating factors, including filgrastim or lenograstim; ^d^P-glycoprotein inhibitors: amiodarone, azithromycin, captopril, carvedilol, clarithromycin, conivaptan, cyclosporine, diltiazem, dronedarone, erythromycin, felodipine, itraconazole, ketoconazole, lopinavir and ritonavir, quercetin, quinidine, ranolazine, ticagrelor, and verapamil

**Online Resource 5. M1 Versus M3 Base Model Parameter Estimates.**

|  | **Definition** | **M1 Base Model (Omit Data <LLOQ)** | | | **M3 Base Model (Include Data <LLOQ)** | | |
| --- | --- | --- | --- | --- | --- | --- | --- |
|  |  | **NONMEM Results**  **OFV=‒883.435** | | **Nonparametric Bootstrap Results** | **NONMEM Results**  **OFV=1560.354** | | **Nonparametric Bootstrap Results** |
|  |  | **Estimate**  **(95% CI^a^)** | **Shrinkage** | **Median Estimate**  **(95% CI^b^)** | **Estimate**  **(95% CI^c^)** | **Shrinkage** | **Median Estimate (95% CI^d^)** |
| CL_1,_ L/h | Linear clearance | 0.0842  (0.0750 to 0.0934) | ‒ | 0.0839  (0.0745 to 0.0918) | 0.118  (0.111 to 0.125) | ‒ | 0.118  (0.109 to 0.130) |
| ALL on CL_1_^e^ | / | −0.569  (‒0.639 to ‒0.499) | ‒ | ‒0.573  (‒0.633 to ‒0.489) | −0.716  (‒0.748 to ‒0.684) | ‒ | ‒0.718  (‒0.751 to ‒0.676) |
| CL_2_, L/h | Clearance associated with time-dependent clearance | 0.462  (0.385 to 0.539) | ‒ | 0.458  (0.372 to 0.570) | 0.368  (0.324 to 0.412) | ‒ | 0.371  (0.333 to 0.418) |
| V_1_, L | Volume of distribution in central compartment | 6.10  (5.86 to 6.34) | ‒ | 6.11  (5.85 to 6.37) | 6.64  (6.32 to 6.96) | ‒ | 6.62  (6.34 to 6.92) |
| k_des_, h^-1^ | Decay coefficient associated with time-dependent clearance | 0.114  (0.0832 to 0.145) | ‒ | 0.111  (0.0784 to 0.169) | 0.0322  (0.0229 to 0.0415) | ‒ | 0.0315  (0.0219 to 0.0426) |
| ALL on k_des_^e^ | / | ‒0.950  (‒0.962 to ‒0.938) | ‒ | ‒0.949  (‒0.964 to ‒0.931) | ‒0.830  (‒0.879 to ‒0.781) | ‒ | ‒0.824  (‒0.867 to ‒0.742) |
| Q, L/h | Intercompartment clearance | 0.117  (0.0798 to 0.154) | ‒ | 0.113  (0.0889 to 0.188) | 0.0398  (0.0343 to 0.0453) | ‒ | 0.0405  (0.0346 to 0.0485) |
| V_2_, L | Volume of distribution in peripheral compartment | 8.19  (6.07 to 10.3) | ‒ | 8.20  (6.38 to 11.2) | 5.47  (4.91 to 6.03) | ‒ | 5.45  (3.22 to 8.61) |
| CL_1_ ω^2^ (%CV) | Variance-covariance matrix of the interindividual effects in CL_1_ | 0.243 (49.3)  (0.134 to 0.352) | 19.3 | 0.239 (48.9)  (0.127 to 0.353) | 0.258 (50.8)  (0.216 to 0.300) | 14.6 | 0.264 (51.4)  (0.172 to 0.381) |
| CL_2_ ω^2^ (%CV) | Variance-covariance matrix of the interindividual effects in CL_2_ | 0.520 (71.1)  (0.302 to 0.738) | 30.7 | 0.499 (70.6)  (0.269 to 0.771) | 0.601 (77.5)  (0.490 to 0.712) | 20.2 | 0.586 (76.6)  (0.352 to 1.14) |
| V_1_ ω^2^ (%CV) | Variance-covariance matrix of the interindividual effects in V_1_ | 0.183 (42.8)  (0.151 to 0.215) | 12.4 | 0.183 (42.8)  (0.152 to 0.212) | 0.206 (45.4)  (0.179 to 0.233) | 13.5 | 0.202 (44.9)  (0.165 to 0.242) |
| k_des_ ω^2^ (%CV) | Variance-covariance matrix of the interindividual effects in k_des_ | 0.184 (42.9)  (0.0915 to 0.277) | 60.7 | 0.189 (43.5)  (0.0980 to 0.346) | 0.255 (50.5)  (0.181 to 0.329) | 59.1 | 0.247 (49.7)  (0.129 to 0.447) |
| CL_1_‒V_1_ ω, covariance | / | 0.183^f^  (0.125 to 0.241) | ‒ | 0.179  (0.126 to 0.244) | 0.216^g^  (0.187 to 0.245) | ‒ | 0.214  (0.167 to 0.274) |
| CL_1_‒CL_2_ ω, covariance | / | 0.135^f^  (0.0401 to 0.230) | ‒ | 0.140  (0.0374 to 0.248) | 0.327^g^  (0.280 to 0.374) | ‒ | 0.317  (0.220 to 0.437) |
| CL_2_‒V_1_ ω, covariance | / | 0.178^f^  (0.107 to 0.249) | ‒ | 0.183  (0.107 to 0.250) | 0.291^g^  (0.250 to 0.332) | ‒ | 0.278  (0.206 to 0.361) |
| Res Err – NHL^h^ | / | 0.367  (0.323 to 0.411) | 11.2 | 0.367  (0.325 to 0.413) | 0.453  (0.447 to 0.459) | 18.7 | 0.452  (0.400 to 0.502) |
| Res Err – ALL^g^ | / | 0.539  (0.493 to 0.585) |  | 0.540  (0.490 to 0.587) | 0.641  (0.634 to 0.648) | ‒ | 0.634  (0.572 to 0.704) |

Res Prop Err=residual proportional error; SE=standard error.

^a^The 95% CI was manually calculated using the following equation: Estimate ± 1.96 × SE. SE was obtained from the covariance step using the R/S Matrix in NONMEM; ^b^999 of 1000 bootstraps had successful minimization, with 89 of those bootstraps with estimates near boundary; hence, a total of 910 bootstraps were included in the calculation of the 95% CI; ^c^the 95% CI was manually calculated using equation: Estimate ± 1.96 × SE. SE was obtained from the covariance step using the S Matrix in NONMEM (R and R/S Matrix had unsuccessful covariance steps; ^d^640 of 1000 bootstraps had successful minimization, with 7 of those bootstraps with estimates near boundary; hence, a total of 633 bootstraps were included in the calculation of the 95% CI; ^e^ALL effects on CL_1_ and k_des_ accounted for the different diseases (ie, ALL vs NHL) and/or bioanalytical assay methods (ie, HPLC/MS/MS for ALL studies and ELISA for NHL studies); ^f^correlation was estimated to be 87.0% for CL_1_−V_1_, 38.1% for CL_1_−CL_2_, and 57.7% for CL_2_−V_1_; ^g^correlation was estimated to be 93.8% for CL_1_−V_1_, 83.0% for CL_1_−CL_2_, and 82.9% for CL_2_-V_1_; ^h^2 residual errors were included in the model for the type of disease (ie, ALL vs NHL) and/or bioanalytical assay method (ie, HPLC/MS/MS for ALL studies and ELISA for NHL studies).

**Online Resource 6. Visual predictive checks M3 final model.**


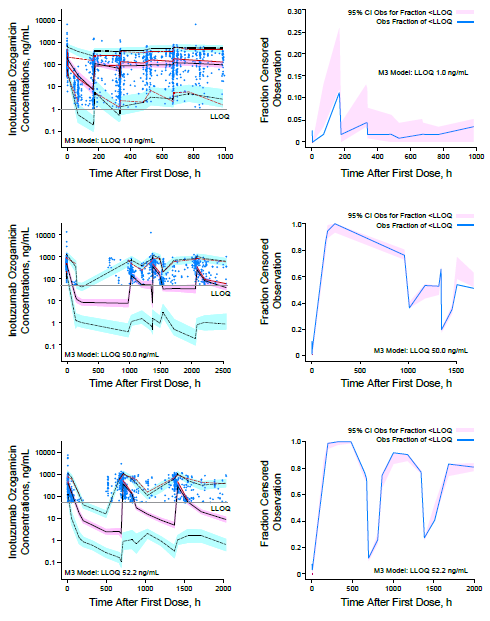


For plots on the left, blue circles are observed data. Red lines are the 50th (solid), 5th (dash), and 95th (dash) percentiles of observed data. Gray lines are LLOQ values. The 50th, 5th, and 95th percentiles of simulated data (black lines) are calculated for each time bin. The 95% CIs are shown by pink shaded areas for the simulated 50th percentile and in shaded blue areas for the simulated 5th and 95th each percentile. For plots on the right, the blue solid lines are the fraction of observed data <LLOQ and the pink shaded areas are the 95% CI for fraction of observed data <LLOQ. The full x-axis ranges are not shown for better visualization.


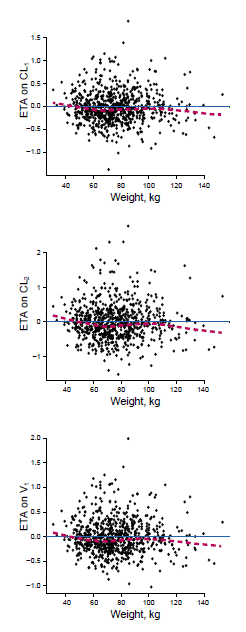
**Online Resource 7. Final model empirical Bayes estimate of the interindividual random effect in a pharmacokinetic parameter versus A) baseline weight and B) gender.**

**A)**

The black dots are the baseline individual observed values. The blue solid lines are the reference line (y=0) and the red dotted lines are the locally weighted scatterplot smoothing trend line (LOESS). Data are represented on the natural number scale.


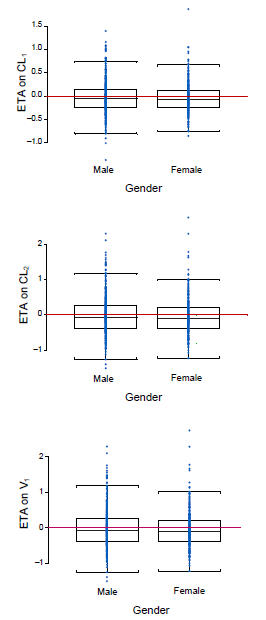
**B)**

Medians for each group are represented by a black solid line, a red line shows the reference line (y=0), and blue circles are individuals ETAs. Data are represented on the natural number scale.


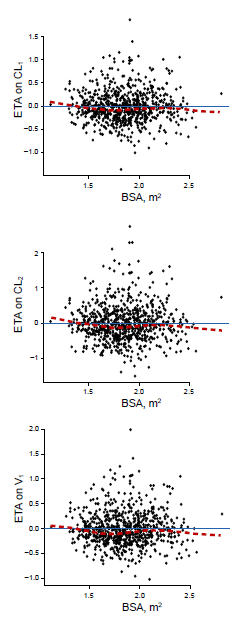
**Online Resource 8. Final model empirical Bayes estimate of the interindividual random effect in a pharmacokinetic parameter versus baseline body surface area.**

The black dots are the baseline individual observed values. The blue solid lines are the reference line (y=0) and the red dotted lines are the locally weighted scatterplot smoothing trend line (LOESS). Data are represented on the natural number scale.

**Online Resource 9. A) Linear clearance and B) final model empirical Bayes estimate of the interindividual random effect on the decay coefficient associated with time-dependent clearance versus rituximab administered with inotuzumab ozogamicin.**

**A)**

**
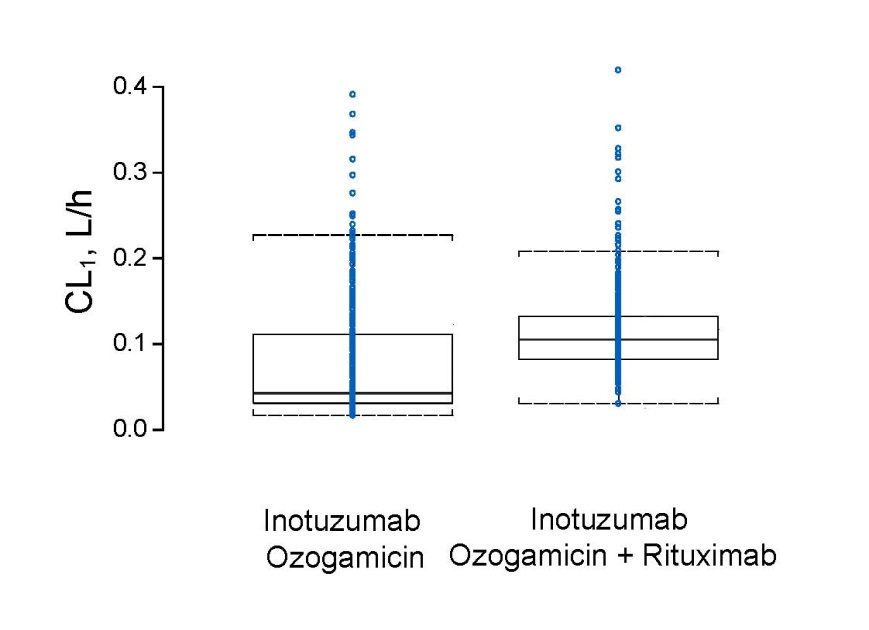
**

**B)**

**
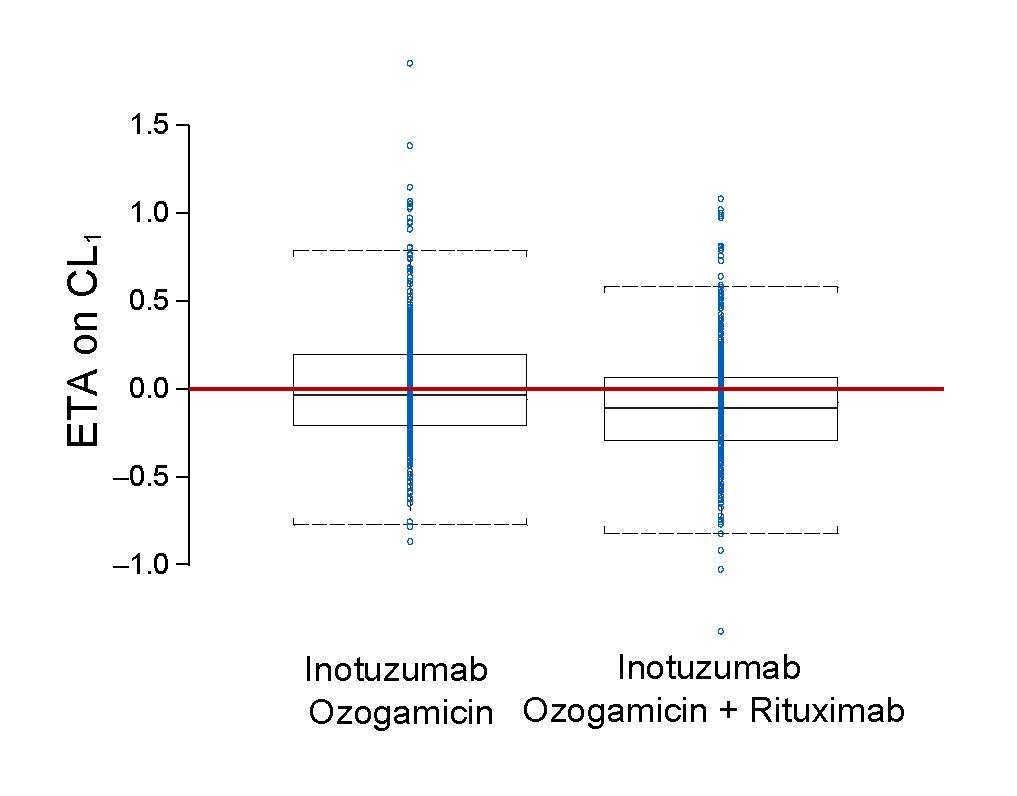
**

Medians for each group are represented by a black solid line, a red line shows the reference line (y=0), and blue circles are individuals ETAs. Data are represented on the natural number scale.
